# Investigation of Canine-Mediated Human Rabies Death, Haiti, 2015

**DOI:** 10.3201/eid2401.161555

**Published:** 2018-01

**Authors:** Cuc H. Tran, Melissa D. Etheart, Lesly L. Andrecy, Pierre Dilius Augustin, Maxwell Kligerman, Kelly Crowdis, Paul Adrien, Amber Dismer, Jesse D. Blanton, Max Millien, Ryan M. Wallace

**Affiliations:** Centers for Disease Control and Prevention, Atlanta, Georgia, USA (C.H. Tran, M. Kligerman, A. Dismer, J.D. Blanton, R.M. Wallace);; US Centers for Disease Control and Prevention, Port au Prince, Haiti (M.D. Etheart);; Ministère de la Santé Publique et de la Population, Delmas, Haiti (L.L. Andrecy, P. Adrien);; Ministère de l'Agriculture, des Resources Naturelles et du Développement Rural, Port au Prince (P.D. Augustin, M. Millien);; Christian Veterinary Mission, Port au Prince (K. Crowdis)

**Keywords:** rabies, Haiti, deaths, canine-mediated, human, viruses, dogs, vaccination, vaccines

## Abstract

In Haiti, an investigation occurred after the death of a 4-year-old girl with suspected rabies. With tips provided by community members, the investigation led to the identification of 2 probable rabies-related deaths and 16 persons bitten by rabid dogs, 75% of which chose postexposure prophylaxis. Community engagement can bolster rabies control.

Haiti is one of the few countries in the Western Hemisphere where canine rabies control has not successfully eliminated canine-mediated human rabies deaths (*1*). During 2010–2012, an average of 4 canine and 7 human rabies cases were reported annually in Haiti; however, this rate is widely recognized as an underestimate of the true burden (*2*‒*4*).

On September 22, 2015, public health officials of Haiti requested assistance with the investigation of a suspected human rabies death in Platfon, a rural part (human population 769) of the Gonaives Arrondissement. The case involved a 4-year-old girl who was bitten on the stomach by a dog 3 months before the onset of illness in late August. She had clinical symptoms consistent with rabies before death: hydrophobia, agitation, and localized paralysis. The child was not taken to the hospital for treatment after the bite or during her illness and died at home on September 8. In response to this probable human rabies case (according to the World Health Organization case definition) (*1*) and reports of increased rabies-like illnesses among dogs in the community, an international multiagency team was assembled to identify potential additional persons bitten for postexposure prophylaxis, conduct a dog vaccination campaign, and characterize healthcare-seeking and dog ownership behaviors.

We identified 4 additional persons bitten by the same dog responsible for the initial probable rabies case. During the investigation, community members requested the team investigate 2 additional events of suspected rabies transmission. The first event involved 10 persons bitten by a dog in the city of Gonaives (Figure, panel A). Brain samples from the dog were laboratory-confirmed positive for rabies virus. The second event involved 3 persons bitten by a dog, 1 of which subsequently died in July. In total, 2 probable human rabies deaths and 16 bitten persons were identified during the 3-week investigation (Figure, panel B). Although educational outreach and postexposure prophylaxis were offered at no cost to all persons bitten, 25% (n = 4) refused vaccination because of religious beliefs. Their current health status is unknown.

**Figure:**
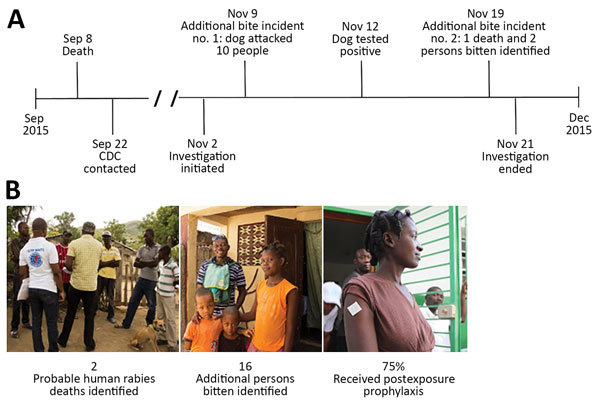
Investigation for probable cases of canine rabies transmission to humans, Haiti, 2015. A) Timeline of investigation and B) outcomes. CDC, Centers for Disease Control and Prevention.

We surveyed 82 dog owners (mean 35.6 [range 18–60] years of age) attending the canine vaccination campaigns in Platfon and the city of Gonaives. The owners reported that 24 (4.3%) of 559 persons living in their households (median persons/household 6.9), including themselves, were bitten by dogs within the past year. Although 70% of survey respondents were aware that rabies can be transmitted through animal bites, only 53% knew that these bites could result in death. Approximately 83% of respondents stated they would seek medical care after an exposure but reported barriers to obtaining treatment: financial obstacles associated with transportation and treatment (47%) and absence of a local medical facility and trained personnel in the community (28%). Respondents stated it took 29 (range 5–180) minutes on average to travel to the nearest medical facility.

We also conducted animal health screenings during the vaccination campaign and estimated the local dog population (*5*) to assess vaccination coverage level. No animals were exhibiting signs consistent with rabies at the time of the investigation. A 2-day count identified 41 dogs, 21 of which had evidence of vaccination from our campaign (51.2% coverage). We estimated 87 dogs in this community and a 9:1 human-to-dog ratio, which is more similar to the ratio in Africa than Latin America, where the human-to-dog ratio is lower (*6*,*7*). Dog owners reported this was the first mass vaccination campaign available to them in the past 5 years. Given Platfon’s remoteness, we believe many of these dogs were never vaccinated before this response.

Our investigation indicates that the rabies burden in Haiti is much greater than detected through passive surveillance because of the lack of healthcare-seeking behavior among persons bitten by dogs. We estimate the rabies-associated mortality rate as 0.67 cases/100,000 population, which is much higher than Haiti’s health facility‒based surveillance system estimates (average 0.07 cases/100,000 population) (*2*–*4*,*8*). The 2 probable human rabies cases and 16 persons bitten identified in this study were not originally reported to health officials because they did not seek care for their exposures and, thereby, were not captured in the national surveillance system.

Furthermore, this underreporting negatively affects proper allocation and stockpiling of nationally procured rabies biologics for animals. Intermittent dog vaccination campaigns are insufficient to break the cycle of rabies transmission. Sustained campaigns targeting 70%–80% of the dog population will be necessary to eliminate canine rabies (*1*,*9*). Anecdotal evidence suggests that social mobilization campaigns to increase healthcare-seeking behavior might be effective; most of the cases investigated in our study were reported by a community member in Platfon who alerted health officials after attending a rabies educator’s workshop. We hope these findings will prompt a discussion for improvements in rabies prevention and control measures that empower community members with knowledge to notify officials of suspected rabies transmission events.
